# PEtab-GUI: a graphical user interface to create, edit, and inspect PEtab parameter estimation problems

**DOI:** 10.1093/bioinformatics/btag106

**Published:** 2026-03-05

**Authors:** Paul J Jost, Frank T Bergmann, Daniel Weindl, Jan Hasenauer

**Affiliations:** Bonn Center for Mathematical Life Sciences, University of Bonn, Bonn 53115, Germany; LIMES Life and Medical Sciences Institute, University of Bonn, Bonn 53115, Germany; BioQUANT, Heidelberg University, Heidelberg 69120, Germany; Bonn Center for Mathematical Life Sciences, University of Bonn, Bonn 53115, Germany; LIMES Life and Medical Sciences Institute, University of Bonn, Bonn 53115, Germany; Bonn Center for Mathematical Life Sciences, University of Bonn, Bonn 53115, Germany; LIMES Life and Medical Sciences Institute, University of Bonn, Bonn 53115, Germany

## Abstract

**Motivation:**

Parameter estimation is a cornerstone of data-driven modeling in systems biology. Yet, constructing such problems in a reproducible and accessible manner remains challenging. The PEtab format has established itself as a powerful community standard to encode parameter estimation problems, promoting interoperability and reusability. However, its reliance on multiple interlinked files—often edited manually—can introduce inconsistencies, and new users often struggle to navigate them. Here, we present PEtab-GUI, an open-source Python application designed to streamline the creation, editing, and validation of PEtab problems through an intuitive graphical user interface. PEtab-GUI integrates all PEtab components, including SBML models and tabular files, into a single environment with live error checking and customizable defaults. Interactive visualization and simulation capabilities enable users to inspect the relationship between the model and the data. PEtab-GUI lowers the barrier to entry for specifying standardized parameter estimation problems, making dynamic modeling more accessible, especially in educational and interdisciplinary settings.

**Availability and implementation:**

PEtab-GUI is implemented in Python, open-source under a 3-Clause BSD license. The code, designed to be modular and extensible, is hosted on https://github.com/PEtab-dev/PEtab-GUI, available as a Zenodo repository at https://doi.org/10.5281/zenodo.15355752, and can be installed from PyPI.

## Introduction

Data-driven mathematical models are integral to understanding complex biological systems and processes. Yet, parameters of such models often remain unknown, necessitating parameter estimation (Villaverde *et al.* 2022, Armistead *et al.* 2024, Burbano de Lara *et al.* 2024). Established and calibrated data-driven mathematical models provide a key resource in further investigation of biological systems, from experimental design to model prediction (Hass *et al.* 2019, Malik-Sheriff *et al.* 2020).

Parameter estimation is nowadays supported by a growing spectrum of tools. However, workflows are often fragmented and difficult to reproduce or adapt (Tiwari *et al.* 2021). Indeed, until the introduction of the parameter estimation table (PEtab) format (Schmiester *et al.* 2021), most tools came with their own input formats, making it harder to switch between them and to reproduce or reuse results. PEtab has successfully bridged the gap between multiple such toolboxes vastly improving the reproducibility and reusability of parameter estimation problems in systems biology. It is supported by multiple toolboxes across various programming languages (Hoops *et al.* 2006, Raue *et al.* 2015, Fröhlich et al. 2021, Schälte *et al.* 2023, Persson *et al.* 2025).

PEtab enables the specification of parameter estimation problems based on standardized tabular files and model specification standards (i.e. SBML (Keating *et al.* 2020)). Yet, despite its many advantages, PEtab’s reliance on a coordinated set of interrelated tabular and model files can itself be a source of error for inexperienced users. For example, renaming an observable identifier will have implications on three of five tabular files, warranting changes there. Additionally, allowed table attributes may not be known by heart, slowing down the editing process. Keeping track of all interconnections manually while constructing the PEtab files separately through conventional editors (e.g. Microsoft Excel, Numbers) makes the process arduous and error prone. Programmatically generating PEtab files poses a high entry barrier, since it demands both an understanding of the file structure and the programming skills to translate problems into the PEtab format. Thus, there is a need to improve the accessibility of the PEtab format, facilitating entry to data-driven mathematical modeling and parameter estimation for a larger group of scientists.

Here, we present PEtab-GUI, a Python-based graphical user interface to aid in creating, editing, and inspecting parameter estimation problems defined in the PEtab format. The application allows combined editing of all the PEtab tabular files as well as the SBML file. PEtab-GUI automates the tracking of the aforementioned interconnections. It allows checking the validity of the current state of the PEtab problem, instantaneous checks for edited cells, and inspection of the integrated data. PEtab-GUI is broadly applicable but particularly valuable for scientists new to dynamic modeling and parameter estimation using PEtab. Thus, our application makes PEtab more accessible and provides a further entry point to data-driven mathematical modeling.

### Features

PEtab encodes parameter estimation problems using a collection of files. Following the original definition (Schmiester *et al.* 2021), these files are: the **model file** describes the biological system using the SBML format, which is widely supported and allows reusing existing models without modification. The **condition file** encodes the experimental settings, such as treatments or genetic backgrounds, under which datasets are collected. The **observable file** maps internal model variables to measurable outputs via observation functions. It also defines noise distributions (e.g. normal or Laplace) and their parameters, which can be estimated as part of the modeling process. The **measurement file** contains the actual experimental data and links each data point to its corresponding condition and observable. It supports optional pre-equilibration settings and allows for measurement-specific overrides of observation parameters. One defines the parameters to be estimated and their bounds in the **parameter file**. It can also include prior distributions for use in optimization or Bayesian inference. Optionally, one can specify how to display data and simulation results together, such as time course or dose response plots, in the **visualization file**. Combining all the previously mentioned files, the **PEtab problem file** serves as a central configuration linking all individual PEtab components. It enables flexible combinations of files for reuse or model comparison, using the YAML format.

To facilitate the creation, editing, and inspection of PEtab problems, we designed PEtab-GUI as a graphical user interface that aids both new and experienced modelers in systems biology (Figure S1, available as supplementary data at *Bioinformatics* online). We designed PEtab-GUI according to feedback from PEtab users and tested it with problems of various sizes from a benchmark collection (Hass *et al.* 2019). The PEtab-GUI features are organized roughly into the aforementioned three major categories, each addressing key bottlenecks in the creation, editing, and inspection of parameter estimation problems.

### Opening, creating, and archiving

The application supports opening both complete and incomplete problems and individual tables, which can also be dragged and dropped into the interface. It automatically detects the type of file that users open and integrates it seamlessly into the current parameter estimation problem. It also resolves a common formatting mismatch between experimental data and the PEtab standard. While PEtab requires each measurement (a specific observable measured at a specific time under specific conditions) to be entered as a separate row, experimental data is often stored in a matrix format, where rows correspond to timepoints or doses and columns to observables. PEtab-GUI allows the user to import such matrices directly, transforming them into the required PEtab format. In addition, it automatically creates a template for the necessary observables and conditions, ensuring that the problem remains PEtab-compliant without requiring manual restructuring. The user can save every table and the SBML model individually, or the whole PEtab problem can be saved to a directory or as a COMBINE archive (Bergmann *et al.* 2014).

### Interactive and intuitive editing

One of the biggest obstacles in creating a PEtab problem from scratch is the need to create up to five different interdependent tables. PEtab-GUI resolves this issue by designing every data table as a dock widget, a freely resizable and movable window whose visibility can be toggled, allowing the user to move single tables to separate screens. Each table supports intuitive operations such as adding or deleting rows and columns and copying and pasting content. To reduce manual effort and potential errors, PEtab-GUI provides context-aware features. Fields with predefined valid entries, such as the “estimate” column in the parameter table, rely on combo boxes to guide selection. Other columns, such as the parameterId in the parameter table, whose options are dynamically specified by the other PEtab components, offer drop-down menus upon editing. PEtab table columns commonly have duplicate values. To speed up the editing process, PEtab-GUI allows editing multiple cells in the same column at once, giving each the same value. Moreover, when a user references a new condition or observable in the measurement table, the software automatically generates a corresponding entry with customizable default values in the appropriate table. The software also includes live cell validation: as users modify entries, the software performs real-time plausibility checks using PEtab-compliant linting tools. This helps catch structural inconsistencies or incomplete data early in the modeling process. Additional conveniences include advanced table filtering and the use of model-derived defaults for values such as parameter bounds or noise specifications.

The SBML model can be viewed in a separate tab, either as the original XML code or auto-converted to the Antimony format (Smith *et al.* 2009), which uses a concise and human-readable syntax for specifying reaction networks. The user can view and edit both versions, which are synchronized with each other.

### Visualization, simulation, and validation

Visualization, simulation, and validation are tightly integrated into the modeling workflow. PEtab-GUI visualizes both measurement and simulation data, facilitating visual validation. The plots can be generated based on different experimental conditions, outputs, or individually through the visualization specification table. The visualization also supports bidirectional highlighting: when users select a data point or condition in the plot, the corresponding entry is highlighted in the table, and vice versa, allowing for seamless navigation between data and model. Since live updates of multiple plots can be computationally demanding, we tested a variety of problems from the PEtab-Benchmark collection. The application was able to open all problems in a matter of seconds, except for a particularly large model (ca. 40 MB), which required >5 minutes. PEtab problems with ∼50 parameters and 300 measurements, which cover the majority of current PEtab problems, were opened and, without noticeable lag, edited and scanned for errors. To maintain performance with larger models, we implemented the possibility to turn off plotting by hiding the plot widget.

To assess model behavior in real time, users can trigger simulations directly within the interface. A single click simulates the current model with BasiCO (Bergmann 2023) using the latest parameter values, and the system automatically overlays the results on the measurement data. This integration provides immediate visual feedback, making it easy to check for available literature values and the general model structure.

For a more hands-on example demonstrating the complete workflow and features, we refer to the documentation on readthedocs, which has additionally been added as supplementary information.

### Software implementation

The application is written in Python 3.11 and installable from PyPI. It is built upon PySide6 (Fitzpatrick 2021), the official Qt binding for Python. This choice offers a robust foundation for cross-platform compatibility while providing the flexibility needed to design an intuitive and responsive user experience tailored to the needs of PEtab problem creation. The code is structured in a Model-View-Controller (MVC) architecture to separate data management, the user interface, and interaction logic. Each of the three components in turn is hierarchically structured for each of the application’s components, such that each component on its own is still using the MVC architecture. This approach not only enhances maintainability and readability but also enables scalable development, as new features can be added with minimal interference with existing components.

## Discussion

The central aim of PEtab-GUI is to lower the barrier to entry for systems biology modeling by facilitating the construction of standardized parameter estimation problems. Modeling pipelines often requires fluency in multiple file formats, coding languages, and simulation tools. By contrast, this application integrates creation, editing, and inspection into an intuitive graphical user interface, enabling users to build parameter estimation problems without requiring extensive programming knowledge or frequent switching between applications. This makes the tool particularly well-suited as an entry point to data-driven mathematical modeling and for educational settings.

Ongoing maintenance plays a key role in determining a tool’s long-term impact and thus its prolonged survival in the scientific community. Using a modular and hierarchical structure not only reduces the maintenance effort but also lays a foundation for future feature expansion. This also includes the necessary extension to future PEtab versions, keeping the support up to date.

A feature not currently supported is the integration of parameter estimation execution, a predominantly programmatic task that may extend beyond the intended scope of a problem formulation tool. This mainly includes the integration of optimizer and simulator choices, as well as evaluation options. Whether such functionality, including job scheduling on computational clusters, should be incorporated into PEtab-GUI or implemented as a separate, dedicated application to maintain clear purposes remains to be determined. Currently, deployment on a cluster is not supported, but as problem formulation itself is a lightweight, non-intensive task, local deployment is well-suited for the GUI’s intended purpose.

While the GUI-centered design provides clarity and structure, it may also impose limits on flexibility when compared to fully script-based modeling. For highly specialized workflows or nonstandard use cases, advanced users may still prefer direct code-level access to PEtab or SBML. This might also be the case when handling large-scale models. The reactivity of the PEtab-GUI is one of its main selling points, but it has the drawback of being cost intensive. In addition, currently, while installation is made possible through pip, it still requires a pre-installed Python environment. For complete beginners, this might provide a hurdle. Enabling bundle installations could be a potential next step to lower the entry barrier further. Other conceivable features include automated parameter import from SBML models, chatbot assistance for guiding users through the problem formulation process (Wehling *et al.* 2025), and a visualization helper enabling modular construction of plots akin to building blocks.

A key strength of the tool lies in its built-in, real-time validation of user inputs. By integrating PEtab linting directly into the table editors, the application shifts error detection to the earliest stages of model development. This proactive approach minimizes silent inconsistencies and encourages best practices throughout the modeling process. It also ensures full compatibility with downstream toolchains, leveraging the PEtab standard to enable smooth and reliable workflows.

## Supplementary Material

btag106_Supplementary_Data

## Data Availability

The open source software is available on GitHub at https://github.com/PEtab-dev/PEtab-GUI and on Zenodo at https://doi.org/10.5281/zenodo.15355752 and on PyPI at https://pypi.org/project/PEtab-GUI/. No new experimental data were generated or analysed in support of this research.

## References

[btag106-B1] Armistead J , Höpfl S, Goldhausen P et al A sphingolipid rheostat controls apoptosis versus apical cell extrusion as alternative tumour-suppressive mechanisms. Cell Death Dis 2024;15:746. 10.1038/s41419-024-07134-2 39397024 PMC11471799

[btag106-B2] Bergmann FT. Basico: a simplified python interface to copasi. JOSS 2023;8:5553. 10.21105/joss.05553

[btag106-B3] Bergmann FT , Adams R, Moodie S et al Combine archive and omex format: one file to share all information to reproduce a modeling project. BMC Bioinformatics 2014;15:369. 10.1186/s12859-014-0369-z 25494900 PMC4272562

[btag106-B4] Burbano de Lara S , Kemmer S, Biermayer I et al Basal met phosphorylation is an indicator of hepatocyte dysregulation in liver disease. Mol Syst Biol 2024;20:187–216. 10.1038/s44320-023-00007-4 38216754 PMC10912216

[btag106-B5] Fitzpatrick M. Create GUI Applications with Python & Qt6 (PySide6 Edition). Amersfoort, Netherlands: Martin Fitzpatrick, 2021.

[btag106-B6] Fröhlich F , Weindl D, Schälte Y et al AMICI: high-performance sensitivity analysis for large ordinary differential equation models. Bioinformatics 2021;37:3676–7. 10.1093/bioinformatics/btab227 33821950 PMC8545331

[btag106-B7] Hass H , Loos C, Raimúndez-Álvarez E et al Benchmark problems for dynamic modeling of intracellular processes. Bioinformatics 2019;35:3073–82. 10.1093/bioinformatics/btz020 30624608 PMC6735869

[btag106-B8] Hoops S , Sahle S, Gauges R et al COPASI – a COmplex PAthway SImulator. Bioinformatics 2006;22:3067–74. 10.1093/bioinformatics/btl485 17032683

[btag106-B9] Keating SM , Waltemath D, König M et al; SBML Level 3 Community members. Sbml level 3: an extensible format for the exchange and reuse of biological models. Mol Syst Biol 2020;16:e9110. 10.15252/msb.20199110 32845085 PMC8411907

[btag106-B10] Malik-Sheriff RS , Glont M, Nguyen TVN et al BioModels—15 years of sharing computational models in life science. Nucleic Acids Res 2020;48:D407–D415. 10.1093/nar/gkz1055 31701150 PMC7145643

[btag106-B11] Persson S , Fröhlich F, Grein S et al Petab.jl: advancing the efficiency and utility of dynamic modelling. Bioinformatics 2025;41:btaf497. 10.1093/bioinformatics/btaf497 40924544 PMC12457741

[btag106-B12] Raue A , Steiert B, Schelker M et al Data2Dynamics: a modeling environment tailored to parameter estimation in dynamical systems. Bioinformatics 2015;31:3558–60. 10.1093/bioinformatics/btv405 26142188

[btag106-B13] Schälte Y , Fröhlich F, Jost PJ et al pyPESTO: a modular and scalable tool for parameter estimation for dynamic models. Bioinformatics 2023;39:btad711. 10.1093/bioinformatics/btad711 37995297 PMC10689677

[btag106-B14] Schmiester L , Schälte Y, Bergmann FT et al PEtab—interoperable specification of parameter estimation problems in systems biology. PLoS Comput Biol 2021;17:e1008646. 10.1371/journal.pcbi.1008646 33497393 PMC7864467

[btag106-B15] Smith LP , Bergmann FT, Chandran D et al Antimony: a modular model definition language. Bioinformatics 2009;25:2452–4. 10.1093/bioinformatics/btp401 19578039 PMC2735663

[btag106-B16] Tiwari K , Kananathan S, Roberts MG et al Reproducibility in systems biology modelling. Mol Syst Biol 2021;17:e9982. 10.15252/msb.20209982 33620773 PMC7901289

[btag106-B17] Villaverde AF , Pathirana D, Fröhlich F et al A protocol for dynamic model calibration. Brief Bioinform 2022;23:bbab387. 10.1093/bib/bbab387 34619769 PMC8769694

[btag106-B18] Wehling L , Singh G, Mulyadi AW et al Talk2Biomodels: AI agent-based open-source LLM initiative for kinetic biological models. BMC Bioinformatics 2025;26:276. 10.1186/s12859-025-06310-1 41254502 PMC12625589

