# Supplementary material for: PEtab-GUI: a graphical user interface to create, edit, and inspect PEtab parameter estimation problems
Source: Bioinformatics. 2026 Mar 5;42(3):btag106. doi: 10.1093/bioinformatics/btag106 (PMC13012594; doi:10.1093/bioinformatics/btag106)
Supplement: btag106_Supplementary_Data [file btag106_supplementary_data.pdf]

Supplementary Information to:

PEtab-GUI: A graphical user interface to create, edit and inspect PEtab parameter estimation problems

Paul J Jost[1,2], Frank T Bergmann[3], Daniel Weindl[1,2], and Jan Hasenauer[1,2,*]

[1]Bonn Center for Mathematical Life Sciences, University of Bonn, Bonn 53115, Germany
[2]Life and Medical Sciences (LIMES) Institute, University of Bonn, Bonn 53115, Germany
[3]BioQUANT, Heidelberg University, Heidelberg 69120, Germany

Contents

1 Supplementary Figures **2**

2 Tutorial and Next Steps from Documentation **2**

1 Supplementary Figures

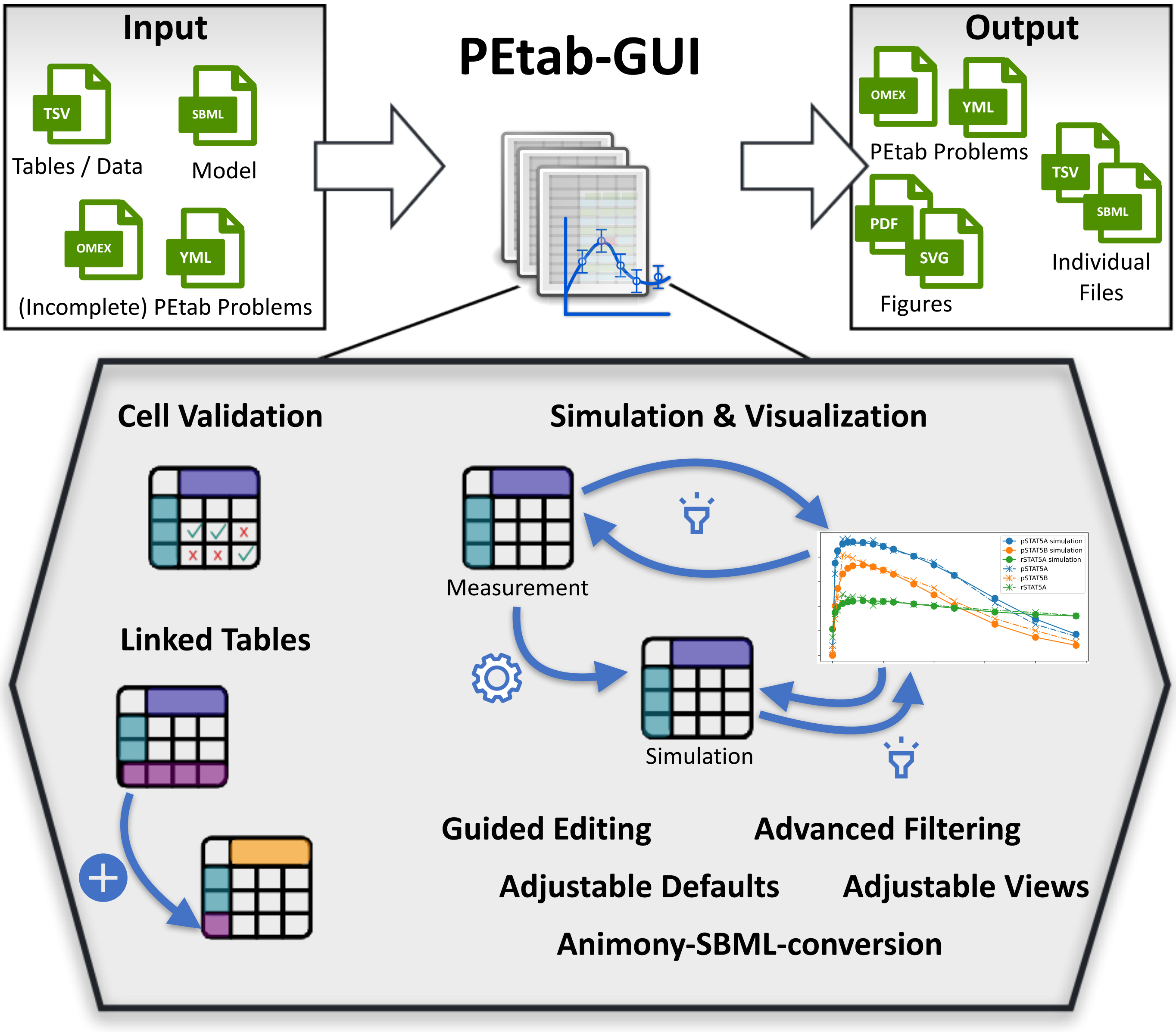

Figure S1: Schema of the PEtab-GUI functionality. Individual tables, SBML models, data files as well as (in-)complete PEtab problems can be imported. Multiple features, such as cell validation, default values, and automated addition of new ID's, help the user to create, edit, or inspect their PEtab problem, which can then be saved in different formats again. Visualizations can also be exported separately.

2 Tutorial and Next Steps from Documentation

PEtab-GUI Tutorial

This tutorial provides a comprehensive guide to using PEtab-GUI for creating and managing PEtab parameter estimation problems for systems biology models.

Introduction

PEtab-GUI is a graphical user interface for the PEtab format, which is a standardized way to specify parameter estimation problems in systems biology. This tutorial will guide you through the entire workflow of creating a parameter estimation problem using PEtab-GUI.

Getting Started

Installation

Before you begin, make sure you have PEtab-GUI installed. You can install it directly from PyPI using pip:

```
pip install petab-gui
```

Alternatively, you can install it from the GitHub repository by following these steps:

1. Clone the repository:

   ```
   git clone https://github.com/PEtab-dev/PEtab-GUI.git
   ```

2. Install using pip:

   ```
   cd PEtab_GUI
   pip install .
   ```

Launching the Application

To start PEtab-GUI, run the following command:

```
petab_gui
```

If you want to open an existing PEtab project, you can specify the path to the YAML file:

```
petab_gui path/to/your/project.yaml
```

The Main Window

When you first launch **PEtab-GUI**, you'll see the main window as shown below:

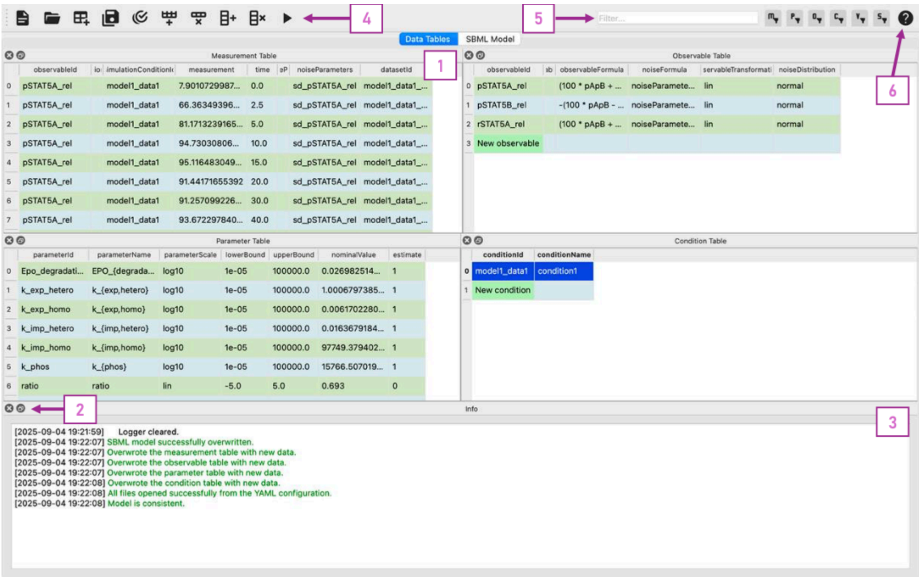

Figure 1: **PEtab-GUI Main Window**: (1) Every Table is in its own dockable panel. Using the buttons in (2) you can get each widget as a separate window or close it entirely. To reopen it, use the `&View` menu in the menu bar. (3) The `Info` widget shows log messages and clickable documentation links. Here you will be informed about deleted lines, potential validation problems and more. (4) The toolbar provides quick access to common actions like opening/saving files, table modification, and model simulation. (5) The filter allows you to only look at specific rows. The filter buttons to the right let you select in which tables the filter should be applied. (6) If you are unsure what to do, you can enter the **Tutorial Mode** by clicking the question mark icon in the toolbar. This will allow you to click different widgets or columns in the tables to get more information about their purpose.

The interface is organized into several key areas:

- **Menu Bar**: At the top, providing access to `&File`, `&Edit`, `&View`, and `&Help`. These items allow you to edit your PEtab problem and navigate the application. Most notably, the `&View` menu allows you to toggle the

visibility of the different panels.

- **Toolbar**: Below the menu bar, offering quick access to common actions like opening/saving files, table modification, and model simulation.
- **Main Window**:

  The main window of the application can be categorized into two main sections that can be selected via tab navigation:

  - **Data Tables** (left tab): Six dockable table panels, each corresponding to a PEtab table (see also the PEtab Documentation):
    - `Measurement Table`: Define experimental observations → See: `measurement-table`
    - `Observable Table`: Specify the formulas and noise models → See: `observable-table`
    - `Visualization Table`: Assign plotting preferences → See: `visualization-table`
    - `Parameter Table`: Set parameter IDs, bounds, and scales → See: `parameter-table`
    - `Condition Table`: Describe experimental conditions → See: `condition-table`
    - `Info` panel: Displays log messages and clickable documentation links
    - `Measurement Plot` panel: At the bottom, visualizes the measurement data based on your current model. → See: `visualization-table`
  - **SBML Model** (right tab): A built-in editor for creating and editing SBML models. It is split into two synced editors:
    - **SBML Model Editor**: For editing the SBML model directly.
    - **Antimony Editor**: For editing the Antimony representation of the model.

    Changes in these can be forwarded to the other editor, allowing you to work in your preferred format. → See: `sbml-editor`

We can can now start creating a new PEtab problem or edit an existing one. The following sections will guide you through the process of defining and editing your model, experimental conditions, measurements, observables, and parameters. While at each step we will learn about the different panels and how to fill the corresponding tables, it might be helpful to have a look at the PEtab Documentation (tutorial) to get a better understanding of the PEtab format and its requirements.

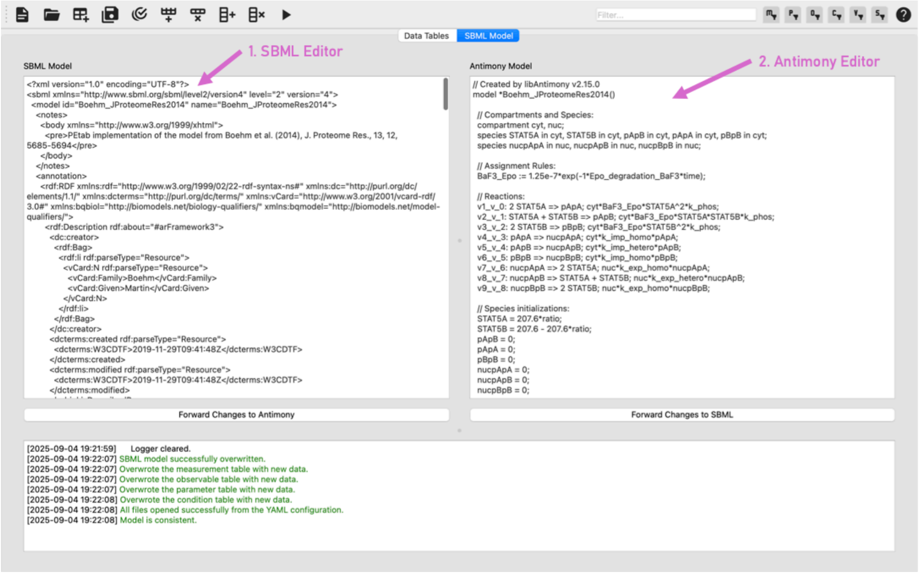

Figure 2: **SBML and Antimony Editors**: The second tab of the GUI application. The SBML editor (1) allows you to edit the SBML model directly, while the Antimony editor (2) provides a more human-readable format. Changes in one editor can be forwarded to the other using the buttons below them.

Quick Start: Your First PEtab Problem

This section provides a complete, hands-on walkthrough to create your first PEtab parameter estimation problem from scratch. You will create a simple conversion model where species A converts to species B, import measurement data for both species in matrix format, and validate the complete problem.

What we'll build: A model describing first-order conversion of species A to species B, with experimental measurements for both species at different time points.

Note

Expected time: 10-15 minutes

Sample data files: Download the example files from the GitHub repository or create them as described below.

Step 1: Launch PEtab-GUI

Start PEtab-GUI from the command line:

```
petab_gui
```

You should see the main window with empty table panels.

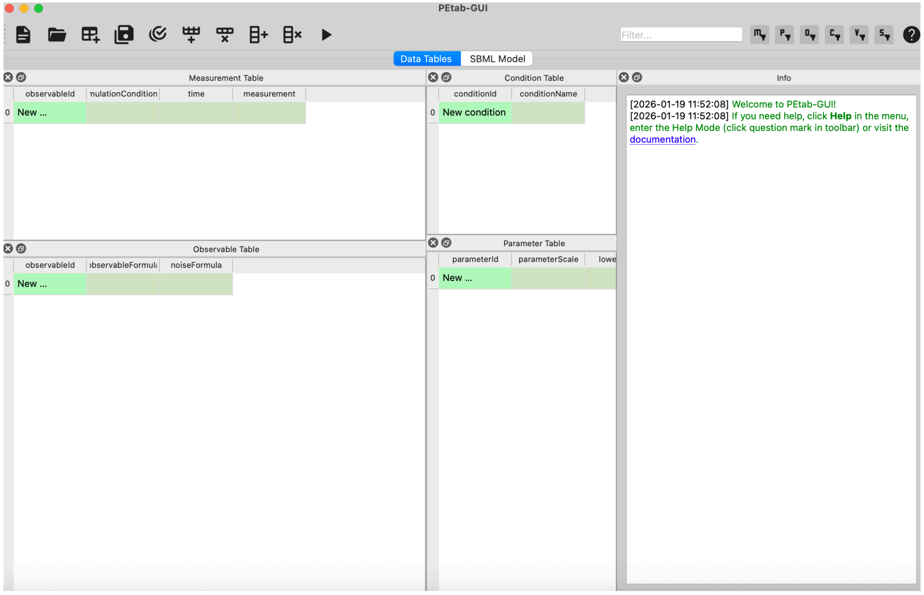

Figure 3: PEtab-GUI on first launch - Data Tables tab showing empty tables ready for a new project.

Step 2: Create the SBML Model

We'll create a simple model using the Antimony editor.

1. Click on the **SBML Model** tab at the top of the main window
2. In the **Antimony Editor** panel (on the right), enter the following model:

```
model *SimpleConversion
  // Reactions:
  conversion: A -> B; k_conversion * A

  // Species initializations:
  A = 10.0  # Initial amount of species A
  B = 0.0   # Initial amount of species B

  // Variable initializations:
  k_conversion = 0.1  # Conversion rate constant
end
```

3. Click the **"Forward changes to SBML"** button below the Antimony editor

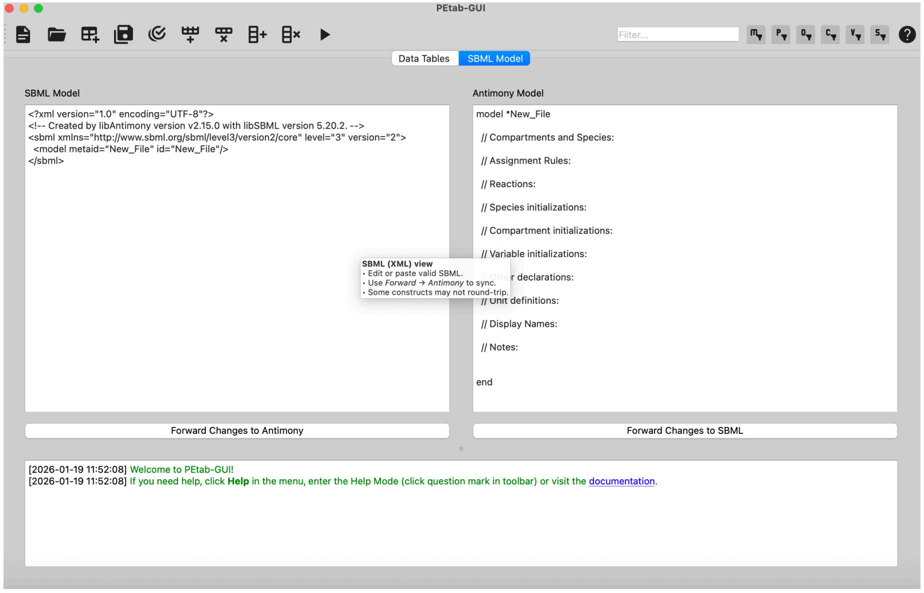

Figure 4: PEtab-GUI on first launch - SBML Model tab with empty editors.

4. You should see the SBML XML representation appear in the **SBML Model Editor** (left panel)

What you should see: The SBML editor should now contain XML code with `<listOfSpecies>`, `<listOfParameters>` and `<listOfReactions>` elements. The Info panel at the bottom might show a message confirming the conversion.

Step 3: Import Measurement Data

Now we'll import experimental measurements.

Option A: Using the provided sample file

1. Download `simple_conversion_measurements.tsv` from the examples folder
2. Switch back to the **Data Tables** tab
3. Drag and drop the TSV file onto the **Measurement Table** panel

Option B: Creating the file yourself

Create a file named `simple_conversion_measurements.tsv` with the following content (a tab-separated values format):

```
time	obs_A	obs_B
0	10.1	0.1
2	8.0	2.1
4	6.8	3.4
```

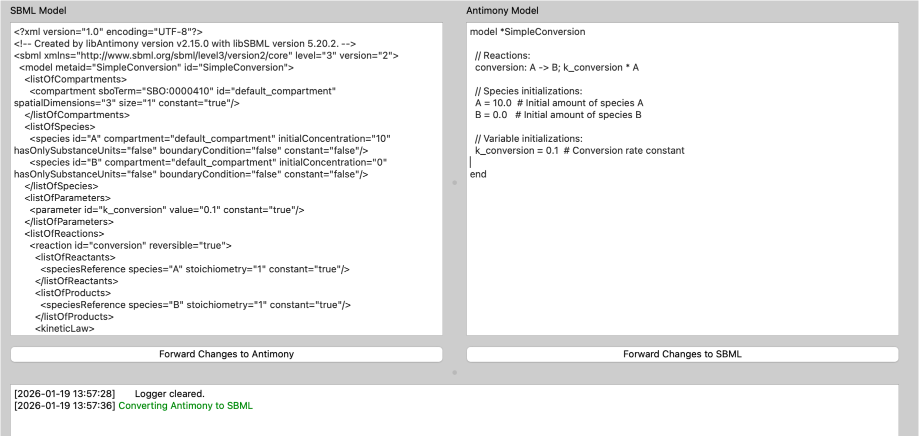

Figure 5: Creating a simple conversion model in Antimony (right panel) and converting it to SBML (left panel).

```
6    5.6 4.6
8    4.4 5.4
10   3.8 6.4
12   3.1 7.2
15   2.3 7.9
18   1.6 8.2
20   1.3 8.5
```

Then drag and drop this file onto the **Measurement Table** panel. When prompted, enter `cond_1` for Simulation Condition. You should now see the measurements imported, the condition and observables created, but some things are marked red. This is the petab linter telling you that some required fields are missing, namely the observable formula, which the GUI can not set automatically. We will fix this in the next steps.

What you should see: The Measurement Table should now contain 20 rows with your measurement data (10 time points for each of the two species). The Info panel will likely show messages about auto-generated observables and conditions.

Step 4: Define the Observable Formulas

PEtab-GUI has automatically created observable entries for both species (`obs_A` and `obs_B`) in the Observable Table, but we need to specify how these observables relate to our model species.

1. Locate the **Observable Table** panel (you may need to scroll or rearrange panels)

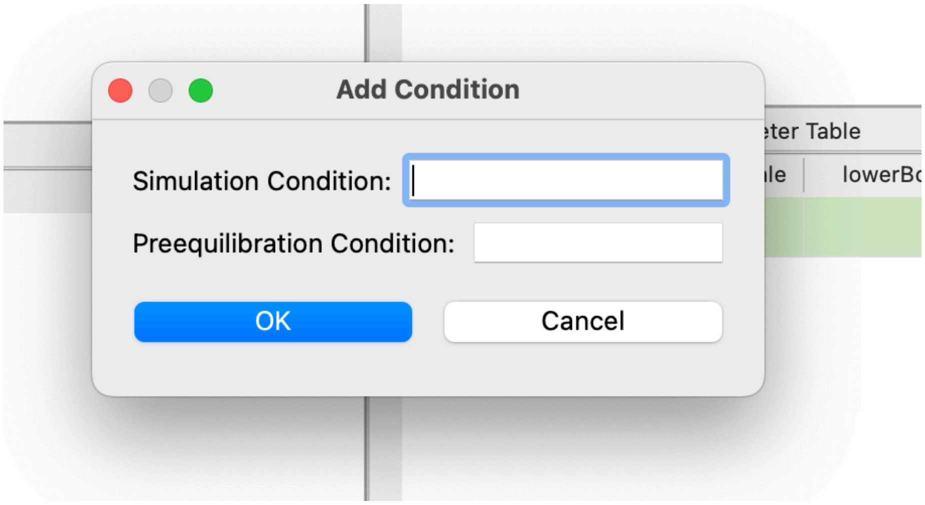

Figure 6: Dialog when uploading data matrix asking for SimulationConditionId.

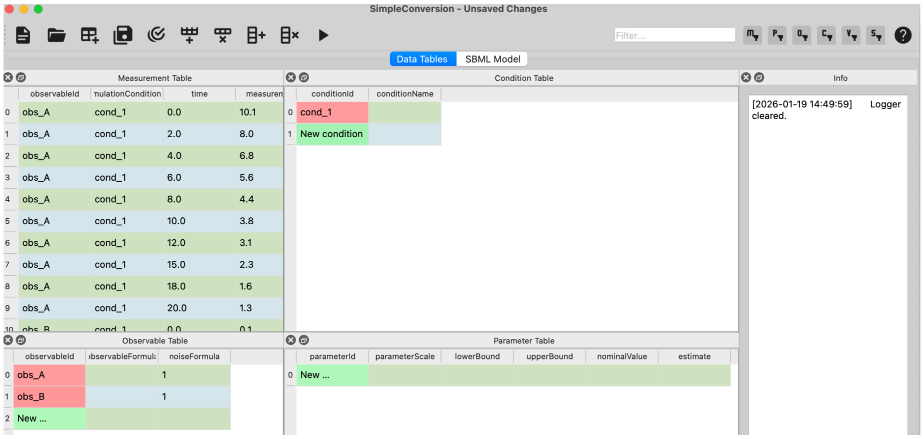

Figure 7: Measurement Table after importing data - 10 time points with measurements for both species A and B.

2. Find the row with `observableId` = `obs_species_A`
3. Click on the `observableFormula` cell (should be empty or have a placeholder)
4. Enter: `A`

   This tells PEtab that the observable directly corresponds to the species A in our model.
5. In the `noiseFormula` cell, enter: `0.5`

   This specifies that measurements have a standard deviation of 0.5 units (normally distributed noise).
6. Now find the row with `observableId` = `obs_species_B`
7. In the `observableFormula` cell, enter: `B`
8. In the `noiseFormula` cell, enter: `0.5`
9. Click Check PEtab in the Toolbar.

You should now see a complete observable table and no more errors in the Info panel.

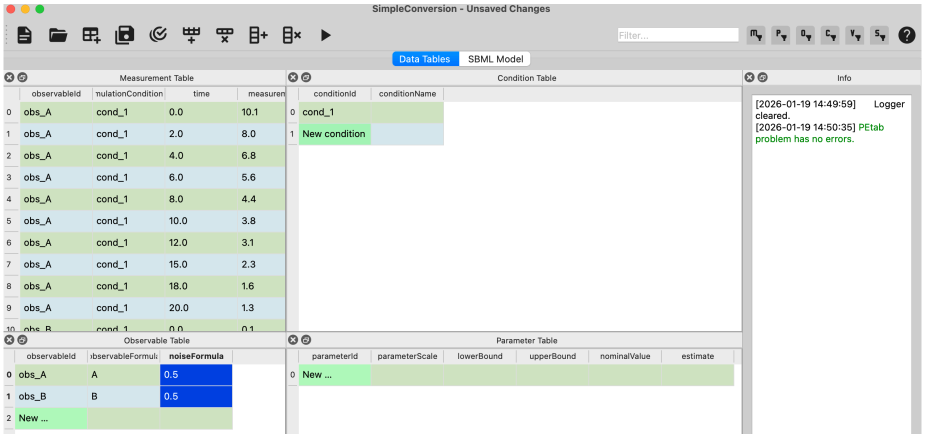

Figure 8: Observable Table with formulas for both species A and B, each with noise standard deviation set to 0.5.

What you should see: The Info panel might show validation messages confirming the observables are now properly defined.

Step 5: Review Auto-Generated Conditions

Switch to the **Condition Table** panel.

What you should see: PEtab-GUI has automatically created an entry for `cond_1` (referenced in your measurements). Since our simple model doesn't require any condition-specific parameter overrides or initial value changes, this table can remain as-is with just the `conditionId` column filled. If you want to rename it, just edit the cell in the **Condition Table** and when subsequently approved, the condition IDs in the **Measurement Table** will be updated accordingly.

Step 6: Configure Parameters for Estimation

Now we specify which parameters should be estimated and their bounds.

1. Switch to the **Parameter Table** panel
2. Click the **"Add Row"** button in the toolbar (or use `&Edit --> Add Row`) or just add it directly by double clicking in the first empty row.
3. Start filling in `k_`, you should automatically be prompted to select `k_conversion` from a dropdown of model parameters.
4. The nominalValue will be taken from your SBML model, the parameterScale should be set to `log10` by default and estimate should set to `1`.
5. You only need to fill out the lower and upper bounds now. Fill in `0.001` and `100` respectively.

Note

We use `log10` scale because rate constants often span several orders of magnitude, and optimization works better in log space. Feel free to use linear scale for parameters as you see fit.

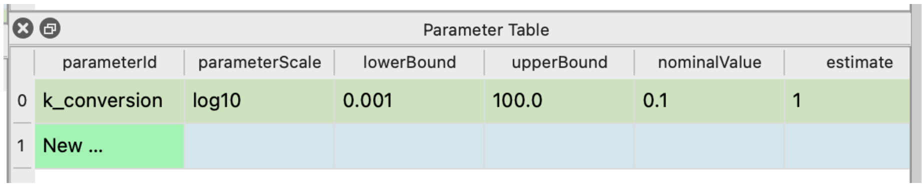
Parameter Table

	parameterId	parameterScale	lowerBound	upperBound	nominalValue	estimate
0	k_conversion	log10	0.001	100.0	0.1	1
1	New ...					

Figure 9: Parameter Table configured for estimating k_conversion with bounds [0.001, 100] on log10 scale.

What you should see: One row in the Parameter Table with all columns filled. Nothing should be colored red anymore.

Step 7: Visualize Your Measurements

Let's see what our measurement data looks like.

1. Make sure the **Data Plot** panel is visible at the bottom (if not, enable it via `&View --> Data Plot`)
2. You should see two plot with time on the x-axis and measurement values on the y-axis, one for obs_A and one for obs_B.

3. If you want to see only one plot with both species, click the cogwheel icon in the Measurement Plot panel and select Group by condition.
4. The plot should show two sets of data points: one for species A (decreasing over time) and one for species B (increasing over time), with about 10 time points each.

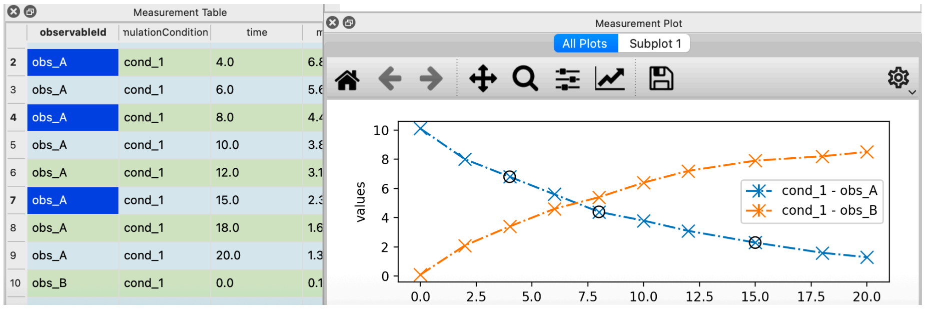

Figure 10: Measurement Plot showing the experimental data - time points vs. measured values for both species A (decreasing) and species B (increasing).

Try this: In the `Measurement Table` select one or multiple rows. The corresponding point(s) in the `Data Plot` will be highlighted. This linking lets you explore and validate your data early on.

Step 8: Run a Simulation

Now let's simulate the model with our current parameter values to see how well it fits the data.

1. In the toolbar, click the **"Simulate"** button (usually has a "play" or "gear" icon)
2. Wait a few seconds for the simulation to complete
3. The **Simulation Table** panel should appear (if not, enable it via `&View --> Simulation Table`)
4. In the **Data Plot** panel - you should now see both your measurements (dots) and the simulation (lines)

What you should see: Two line plots overlaid on your measurement points - one for species A (decreasing) and one for species B (increasing). If you used the exact values from this tutorial, the simulation should match the measurements reasonably well since the data was generated with k_conversion 0.1.

Step 9: Save Your Project

Congratulations! Your PEtab problem is complete. Now let's save it.

1. Go to `&File --> Save As...`
2. Choose a location and filename (e.g., `simple_conversion`)

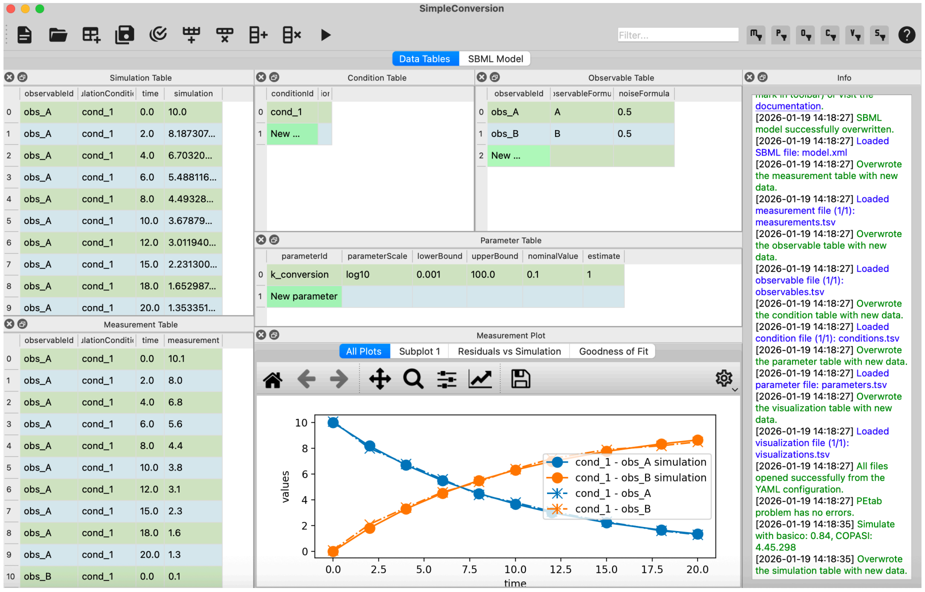

Figure 11: Measurement Plot after running simulation - measurements (dots) and model simulation (lines) are shown together for both species A and B. The conversion rate k_conversion=0.1 provides a good fit to the data.

3. Select the format:
   - **Folder**: Creates a folder with all table/model files with corresponding YAML file (recommended for local editing)
   - **COMBINE Archive (.omex)**: Saves everything in a single compressed archive (recommended for sharing)
4. Click **Save**

What you should see: A success message detailing the folder path. When clicking ok, a `Next Steps` dialog appears with links to relevant documentation.

Congratulations!

You've successfully created your first PEtab parameter estimation problem! You now have:

An SBML model describing the conversion of species A to species B

Experimental measurements for both species A and B linked to the model

Observable and condition definitions for both species

Parameters configured for estimation with appropriate bounds

A validated PEtab problem ready for parameter estimation tools

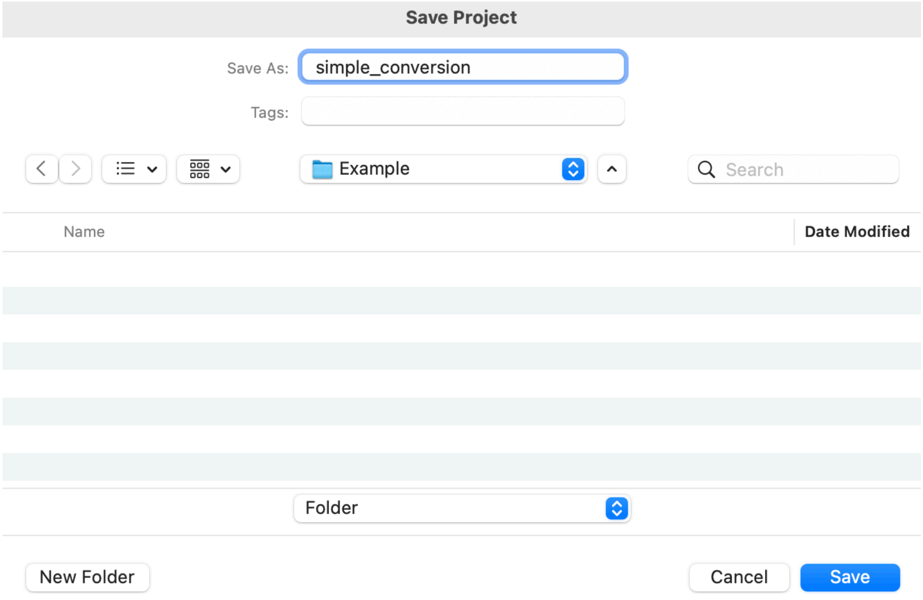

Figure 12: Saving your PEtab problem - choose folder format for easy editing or COMBINE archive for sharing.

Next Steps:

- **Learn more about the abilities of the GUI below on a more high level, specifically**
  - about `matrix-import` for handling experimental data in matrix format (which we used in this tutorial!)
  - the `visualization-table` for customizing plots
  - about `advanced-features` like filtering and batch editing
- Try changing the parameter values and re-running the simulation or dive immediately into parameter estimation using tools like pyPESTO

Using your PEtab problem: The saved YAML file can be used with parameter estimation tools like:

- pyPESTO - Parameter estimation toolbox
- COPASI - Biochemical model simulator and analysis tool
- AMICI - Advanced sensitivity analysis

Opening an Existing PEtab Problem

If you already have a PEtab problem defined in a YAML file or you have your SBML model already, you can open them directly in PEtab-GUI:

1. Through the menu bar, go to `&File --> &Open`. This will open a file dialog, where you can select your YAML file, SBML model file, or any other PEtab-related files.
2. Alternatively, you can drag and drop your YAML file onto the PEtab-GUI window. The application will automatically handle the file and load the relevant data into the interface.
3. If you want to continue working on an existing PEtab problem, you can also use the `&File --> Recent Files` menu to quickly access recently opened projects.

Creating/Editing a PEtab Problem

Since a PEtab problem consists of several components, we will go through the process step by step. The following sections will guide you through creating or editing a PEtab problem using the PEtab-GUI. While there is no strict order in which you have to fill the tables, we will follow a logical sequence that starts with the model definition, followed by measurements, experimental conditions, observables, and parameters.

Creating/Editing an SBML Model

Usually the first step in creating a PEtab problem is to define the underlying SBML model. Independent of whether you are creating a new model or editing an existing one, you are given the choice between editing the model directly in SBML or in the much more readable Antimony and then converting it to SBML.

Need help understanding what an SBML model is?

SBML (Systems Biology Markup Language) is an XML-based format for representing computational models of biological processes. It describes the components of a biological system:

- **Species**: Molecular entities (proteins, metabolites, genes, etc.) that can change over time
- **Reactions**: Processes that transform species (e.g., enzymatic reactions, binding/unbinding events)
- **Parameters**: Constants that define reaction rates, initial concentrations, and other quantities
- **Compartments**: Physical locations where species exist (e.g., cytoplasm, nucleus, extracellular space)

SBML files are typically generated by modeling tools or written programmatically. While SBML is precise and machine-readable, it can be verbose and difficult to read/write manually. That's why PEtab-GUI supports **Antimony**, a human-readable text format that can be easily converted to SBML. If you're new to biological modeling, we recommend starting with Antimony and converting to SBML when needed.

For more information, see the SBML website and the Antimony documentation.

If you are creating a new model, the empty antimony template might help in getting started. Here is a simple example showcasing how species, reactions, and parameters can be defined:

```
model *ExampleModel
  // Reactions
  J0: S1 -> S2 + S3; k1*S1 # Mass-action kinetics
  J1: S2 -> S3 + S4; k2*S2
  // Species initialization
  S1 = 10 # The initial concentration of S1
  S2 = 0  # The initial concentration of S3
  S3 = 3  # The initial concentration of S3
  S4 = 0  # The initial concentration of S4
  // Variable initialization
  k1 = 0.1 # The value of the kinetic parameter from J0.
  k2 = 0.2 # The value of the kinetic parameter from J1.
end
```

Specifying Measurements

Indispensable for parameter estimation problems are the measurements that will be used to fit the model parameters. In PEtab-GUI, you can define these measurements in the `Measurement Table`. While it is possible to create a new measurement table from scratch, it is usually more convenient to import an already existing measurement file. In our experience, most measurements exist in some matrix format. Time-resolved data might have each row corresponding to a time point and each column corresponding to a different observable. Similar can Dose-Response data be structured, where each row corresponds to a different dose. Accounting for these common formats, PEtab-GUI handles opening a CSV or TSV file by checking whether it is a time series, dose-response, or a PEtab measurement file. Simply drag and drop your file into the **Measurement Table** or use the `&File --> &Open` option. In general what we need to specify in the measurement table are:

1. **observableId**: A unique identifier for the observable that this measurement corresponds to. This should match the observable IDs defined in the **Observable Table**.
2. **simulationConditionId**: The condition under which the measurement was taken. You are free to choose a name but it should be consistent with the conditions defined in the **Condition Table**.
3. **time** and **measurement**: The time point and corresponding measurements.

There are a number of optional columns that can be specified, for more details see the PEtab Documentation.

Importing Matrix-Format Measurement Data

One of PEtab-GUI's most powerful features is its ability to automatically convert matrix-format experimental data into PEtab format. This is particularly useful because most experimental data is initially organized in matrix layouts.

Common matrix formats:

- **Time-series data**: Rows = time points, Columns = different conditions or replicates
- **Dose-response data**: Rows = different doses, Columns = different observables or replicates
- **Multi-condition experiments**: Any tabular layout where measurements are organized by experimental variables

Understanding Matrix Format

Matrix format is how experimental data is typically recorded and stored in spreadsheets. Here's an example:

```
time,control,low_dose,medium_dose,high_dose
0,100.5,98.2,101.3,99.7
2,95.3,88.5,82.1,70.4
4,90.8,79.8,67.4,50.2
6,86.2,72.1,55.3,35.8
8,82.0,65.4,45.6,25.6
10,78.3,59.6,37.8,18.4
```

In this format:

- **First column** (`time`): The independent variable (time, dose, etc.)
- **Other columns**: Measurement values for different conditions
- **Column headers**: Become condition names or observable names

This needs to be converted to PEtab's "long format" where each measurement is a separate row with explicit `observableId`, `simulationConditionId`, `time`, and `measurement` columns.

PEtab-GUI handles this conversion automatically!

Step-by-Step: Importing Matrix Data

Let's walk through importing the matrix data shown above.

Step 1: Prepare Your Matrix File

Create a CSV or TSV file with your matrix data. Requirements:

- First row must contain column headers
- First column should be the independent variable (typically `time` or `dose`)
- Remaining columns contain measurement values
- Use clear, descriptive column names (they will become observable IDs)

Step 2: Import the Matrix File

1. Make sure you have an SBML model already loaded (PEtab-GUI needs to know what species exist)

2. In the **Data Tables** tab, locate the **Measurement Table** panel

3. **Drag and drop** your file onto the Measurement Table

   OR

   Use `&File --> &Open` and select your matrix file

4. When prompted, enter the name for the `simulationConditionId` column (e.g., `condition`)

Step 3: Watch the Automatic Conversion

PEtab-GUI will automatically:

1. **Detect** that your file is in matrix format (not already in PEtab format)
2. **Convert** the matrix to PEtab long format:
   - Each cell in the matrix becomes a separate row in the Measurement Table
   - Column names become `simulationConditionId` values (`control`, `low_dose`, etc.)
   - The first column values become `time` values
   - Cell values become `measurement` values
3. **Generate observables**: Create entries in the Observable Table (one per matrix column)
4. **Generate conditions**: Create entries in the Condition Table (as per Prompt)

Step 4: Complete the Observable Definitions

The auto-generated observables need their formulas defined:

1. Switch to the **Observable Table**
2. For each observable (e.g., `obs_A`, `obs_B`), fill in the `observableFormula` column
3. Fill in the `noiseFormula` column (e.g., `0.5` for constant noise, or `0.1*A` for proportional noise)

```
Example Observable Table after completion:
observableId | observableFormula | noiseFormula
obs_A        | A                 | 0.5
obs_A        | A                 | 0.5
obs_A        | A                 | 0.5
obs_B        | B                 | 0.5
```

Step 5: Verify and Visualize

1. Run a lint check to verify everything is correctly configured

2. View the **Measurement Plot** to see all your data visualized
3. Run a simulation to see how your model fits the data

Common Matrix Import Scenarios

Scenario 1: Replicates in Columns

If your matrix has replicates as separate columns:

```
time,sample1_rep1,sample1_rep2,sample1_rep3
0,10.1,9.8,10.3
2,7.9,8.1,7.7
4,6.0,5.8,6.2
```

PEtab-GUI will treat each column as a separate observable. You can:

- Keep them separate with different observable IDs
- Manually merge them by editing the `observableId` column to use the same ID (`sample1`) for all replicates

Scenario 2: Multiple Observables

If your matrix contains different observables:

```
time,protein_A,protein_B,mRNA_A
0,100,50,200
2,95,48,180
4,90,46,160
```

Each column will become a separate observable. Ensure you:

- Map each observable to the correct model species in the `observableFormula`
- Use appropriate noise formulas for each observable type

Scenario 3: Dose-Response Data

For dose-response experiments (rows = doses, no time column):

```
dose,obsA,obsB
0,100,100
0.1,98,95
1.0,85,70
10.0,60,40
```

PEtab-GUI will:

- Use the first column (`dose`) as the independent variable
- Enter a `time` value when prompted
- Create observables for each subsequent column
- Create conditions based on the dose levels

Troubleshooting Matrix Import

Issue: First column not recognized as time

- **Cause**: Column name doesn't match expected patterns
- **Solution**: Ensure first column is named `time`, `dose`, or another clear variable name. You can also manually edit the Measurement Table after import.

Issue: Special characters in column names cause problems

- **Cause**: Column names with spaces, special characters become problematic IDs
- **Solution**: Use simple alphanumeric names with underscores (e.g., `high_dose` instead of `High Dose (mg/L)`)

Tips for Successful Matrix Import

1. **Use clear column names**: These become observable IDs, so make them descriptive but simple
2. **Keep matrix format simple**: Header row + data rows, no extra formatting
3. **One independent variable**: First column should be time, dose, or similar
4. **Consistent data types**: All measurement columns should contain numeric values
5. **Load SBML model first**: PEtab-GUI can better auto-generate formulas if it knows what species exist
6. **Review auto-generated entries**: Always check the Observable and Condition tables after import

Next Steps After Matrix Import:

- Define observable formulas in the Observable Table
- Configure condition-specific parameters if needed
- Add parameter definitions to the Parameter Table
- Run linting to validate the conversion
- Visualize and simulate to verify correctness

Defining Observables

Observables define how model species are mapped to measured quantities. When you create a measurement in the **Measurement Table**, you need to specify which observable it corresponds to. If it is not already defined, PEtab-GUI will automatically create a new observable entry in the **Observable Table**. You will only have to fill out the actual function in the **observableFormula** column, which defines how the observable is calculated from the model species. In the easiest case, this just corresponds to the species

ID, e.g. `S1`. But it could also be a more complex expression like `k_scale * (S1 + S2)`, that even introduces new parameters, e.g. `k_scale`.

In general, we assume that the measurement is subject to some noise. Per default the noise is normally distributed and within the noiseFormula column you can specify the standard deviation of the noise. Again, this formula can be a simple number or a more complex formula introducing new parameters.

For more details, for example on how to change the noise model, see the PEtab Documentation.

Setting Up Experimental Conditions

Experimental conditions define the specific settings under which measurements were taken. Aside from the conditionId column, all other columns are optional. The other columns may either set a parameter to take different values across the conditions or an initial value for a species that is different across conditions (e.g. in case of a dose-response experiment). Just as in the observable table, new conditions can be created automatically when you create a new measurement in the **Measurement Table**.

Setting Up Parameters

The last thing you will want to fill out is the **Parameter Table**. This table defines the parameters that are part of the estimation problem. This includes parameters from the SBML model, observables, and noise models. For every parameter you declare in the estimate column whether it should be estimated during the parameter estimation or not. Additionally you specify lower and upper bounds for the parameter values in the lowerBound and upperBound columns, respectively. If your parameter is not to be estimated, you need to specify a nominalValue. PEtab-GUI aids you in this process by suggesting parameter IDs from the SBML model you might want to add here.

Validation and Inspection

Once you have filled out all the tables, it is important to validate your PEtab problem to avoid errors during parameter estimation. PEtab-GUI supports this through **Visualization and Simulation** and **Linting** features:

Visualization and Simulation

In the `Measurement Plot` panel, you will see a visualization of your measurements. You can click on individual points in the measurement plot to see the corresponding measurement in the `Measurement Table` and vice versa. This

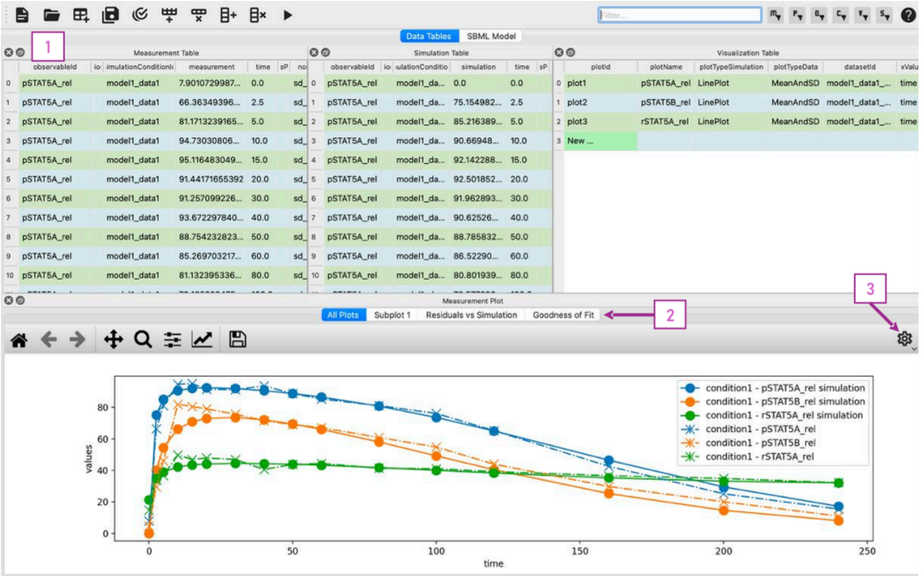

Figure 13: **PEtab-GUI with Visualization and Simulation Panels**: Once you have defined measurements, you can add the **Measurement Plot** to visualize your measurements. You can also add the **Simulation Table** and **Visualization Table** to run simulations and visualize the results. (1) The three tables can be neatly arranged next to each other. (2) Within the measurement panel, you can click on different plots. If you have specified multiple plots, *All Plots* will show every plot specified, followed by tabs for each individual plot. If you have simulations you additionally get a residual plot and a scatterplot of the fit. Through the settings symbol (3) you can change whether you want to plot by observable, condition or defined by the visualization table.

can already help getting an idea of the dynamics of your model and spot potential outliers in your measurements.

Once you have defined all the necessary components, you might want to see whether a specific parameter set leads to a good fit of the model to the measurements. For this you can add two panels to the main interface, the `Simulation Table` and the `Visualization Table`. The **Simulation Table** panel is strictly speaking not part of the PEtab problem definition. Structurally it is the same as the **Measurement Plot** panel, with the sole difference that the column measurement is replaced by simulation. The **Visualization Table** allows you to specify how the measurements (and simulations) should be visualized. In short:

- every plotId corresponds to a specific plot. Rows that have the same plotId will be plotted together.
- You specify your xValues and yValues for each row.
- You can specify additional details, such as offsets and scale. For more details see the PEtab Documentation.

If you dont have simulations yet, you can run a Simulation through the toolbar button, which will automatically fill the **Simulation Table**, running a simulation with the current parameter values and conditions.

If you have simulations, additional plots can be viewed, such as residual plots, as well as goodness-of-fit plots.

Linting

Linting is the process of automatically checking your tables for structural and logical errors during editing.

PEtab-GUI offers two layers of linting support:

- **Partial Linting on Edit**: Whenever you modify a single row in any table, PEtab-GUI will **immediately lint that row** in context. This allows you to catch errors as you build your PEtab problem — such as missing required fields, mismatched IDs, invalid references, or inconsistent units.
- **Full Model Linting**: You can run a complete validation of your PEtab problem by clicking the **lint** icon in the toolbar. This performs a full consistency check across all tables and provides more comprehensive diagnostics.

All linting messages — including errors and warnings — appear in the **Info** panel at the bottom right of the interface. Messages include timestamps, color coding (e.g., red for errors, orange for warnings), and sometimes clickable references or hints.

By using linting early and often, you can avoid many common errors in PEtab problem definition and ensure compatibility with downstream tools.

Advanced Features

PEtab-GUI includes several advanced features to improve your workflow efficiency:

Filtering

The filter bar at the top of the main window allows you to show only specific rows across multiple tables simultaneously:

1. Enter your filter text in the filter input field (e.g., `condition1` or `obs_`)
2. Use the filter buttons to the right to select which tables the filter should apply to
3. The tables will update to show only matching rows
4. Clear the filter to see all rows again

This is particularly useful for:

- Focusing on specific conditions or observables
- Finding and editing related entries across multiple tables
- Debugging issues with specific IDs

Find and Replace

Use the Find & Replace feature for bulk editing:

1. Press `Ctrl+F` (or `Cmd+F` on macOS) to open the Find & Replace bar
2. Enter the text to find
3. Optionally enter replacement text
4. Use ”Find Next” to navigate through matches
5. Use ”Replace” or ”Replace All” for bulk changes

This is useful for:

- Renaming observables or conditions across all tables
- Fixing typos in multiple cells
- Standardizing naming conventions

Multi-Cell Editing

You can edit multiple cells at once:

1. Select multiple cells by clicking and dragging, or by holding `Shift` or `Ctrl` while clicking
2. Type your value and press `Enter`
3. All selected cells will be updated with the same value

This is useful for:

- Setting the same parameter bounds for multiple parameters
- Applying the same noise formula to multiple observables

- Filling in default values quickly

Help Mode / Tutorial Mode

Activate Help Mode to get contextual information:

1. Click the **question mark icon** in the toolbar
2. Click on any widget, table column header, or panel to see context-specific help
3. Help information appears in a popup or the Info panel
4. Exit Help Mode by clicking the question mark icon again

This is useful for:

- Learning what each column means
- Understanding what values are expected in specific fields
- Getting quick reference information without leaving the application

Customizable Table Layout

The table panels are fully dockable and customizable:

1. **Drag panel headers** to rearrange panels
2. **Undock panels** by clicking the undock button (window icon) - panels become separate windows
3. **Close panels** you don't need via the close button or `&View` menu
4. **Restore panels** via `&View` menu
5. **Save your layout** - PEtab-GUI remembers your panel arrangement

This allows you to:

- Arrange panels side-by-side for comparison
- Use multiple monitors effectively
- Hide panels you're not currently using
- Create custom layouts for different tasks

Undo and Redo

PEtab-GUI supports unlimited undo/redo:

- **Undo**: `Ctrl+Z` (or `Cmd+Z` on macOS)
- **Redo**: `Ctrl+Shift+Z` (or `Cmd+Shift+Z` on macOS)

Every table edit is tracked, allowing you to safely experiment and revert changes if needed.

Recent Files

Access recently opened PEtab problems:

- `&File --> Recent Files` shows your recent projects

- Click any entry to quickly reopen a project
- This is useful when working on multiple PEtab problems

Saving Your Project

Once you've set up your parameter estimation problem, and sufficiently validated it, you can save your project. This can be done either as a compressed ZIP file or as a COMBINE archive. You can also save each table as a separate CSV file.

Additional Resources

- PEtab Documentation
- Systems Biology Markup Language (SBML)

Next Steps

Congratulations on completing your PEtab file! Now that you have a standardized parameter estimation problem, you can use various tools to perform parameter estimation, sensitivity analysis, and model simulation. This page provides minimal working examples for the most commonly used tools in the PEtab ecosystem. For a complete list of tools, see the PEtab software support.

Parameter Estimation with pyPESTO

pyPESTO is a Python-based Parameter EStimation TOolbox that provides a unified interface for parameter estimation, uncertainty quantification, and model selection for systems biology models.

Key features:

- Multiple optimization algorithms (local and global)
- Multi-start optimization for local optimizers
- Profile likelihood and sampling for uncertainty analysis
- Native PEtab support

PEtab example notebooks in pyPESTO

- Model import using the PEtab format for a basic optimization of a PEtab problem using pyPESTO.
- AMICI in pyPESTO for a complete workflow of parameter estimation of a PEtab problem using AMICI as simulation engine within pyPESTO.

Minimal working example:

```
import pypesto
import pypesto.petab

# Load PEtab problem
petab_problem = pypesto.petab.PetabImporter.from_yaml("path_to_your_model.yaml")
problem = petab_problem.create_problem()

# Configure optimizer (100 multi-starts)
optimizer = pypesto.optimize.ScipyOptimizer(method='L-BFGS-B')
n_starts = 100
```

```python
# Run optimization
result = pypesto.optimize.minimize(
    problem=problem,
    optimizer=optimizer,
    n_starts=n_starts
)

# Retrieve best parameters
best_params = result.optimize_result.list[0]['x']
print(f"Best parameters: {best_params}")
print(f"Best objective value: {result.optimize_result.list[0]['fval']}")
```

Next steps:

- Perform profile likelihood: pypesto.profile
- Run sampling for uncertainty: pypesto.sample
- Explore different optimizers and settings in pyPESTO, with many more examples in the pyPESTO documentation.

Documentation: https://pypesto.readthedocs.io/

Model Simulation with AMICI

AMICI (Advanced Multilanguage Interface to CVODES and IDAS) provides efficient simulation and sensitivity analysis for ordinary differential equation models.

Disclaimer: AMICI is currently preparing a release v1.0.0, which will have significant changes to the API. The example below corresponds to the current stable release v0.34.2.

Key features:

- C++-based simulation with Python interface
- Fast sensitivity computation via adjoint method
- Symbolic preprocessing for optimized code generation
- Native PEtab support

Minimal working example:

```python
import petab

from amici import runAmiciSimulation
from amici.petab.petab_import import import_petab_problem
from amici.petab.petab_problem import PetabProblem
from amici.petab.simulations import simulate_petab
from amici.plotting import plot_state_trajectories
```

```python
petab_problem = petab.Problem.from_yaml("path_to_your_model.yaml")
amici_model = import_petab_problem(petab_problem, verbose=False)
# Simulate for all conditions
res = simulate_petab(petab_problem, amici_model)
# Visualize trajectory of first condition (indexing starts at 0)
plot_state_trajectories(res["rdatas"][0])
```

Next steps:

- Start to play around with parameters (see this amici example)
- Integrate with pyPESTO for advanced optimization features (see above)

Documentation: https://amici.readthedocs.io/

Model Simulation with COPASI

COPASI (COmplex PAthway SImulator) is a standalone software with a graphical user interface for modeling and simulation of biochemical networks.

Key features:

- Cross-platform GUI application (Windows, macOS, Linux)
- Advanced simulation possibilities (deterministic, stochastic, steady-state)
- User friendly creation and adaptation of SBML models, e.g. introducing events
- Support for parameter estimation and sensitivity analysis

Python Interface:

COPASI also provides the python interface basiCO, which supports the full feature set of PEtab.

```python
from basico import *
import basico.petab
from petab import Problem
import petab.visualize


pp = Problem.from_yaml('./Elowitz_Nature2000/Elowitz_Nature2000.yaml')
sim = basico.petab.PetabSimulator(pp, working_dir='./temp_dir/')
df = sim.simulate()
petab.visualize.plot_problem(pp, simulations_df=df)
```

see here for an example notebook.

Documentation: https://copasi.org/Support/User_Manual/ and https://basico.readthedocs.io/

Parameter Estimation with PEtab.jl

PEtab.jl is a Julia library for working with PEtab files, offering high-performance parameter estimation with automatic differentiation.

Key features:

- High-performance Julia implementation
- Automatic differentiation for fast gradient computation
- Support for ODE and SDE models
- Native integration with Optimization.jl

Minimal working example:

```julia
using PEtab

# Import PEtab problem from YAML
model = PEtabModel("your_model.yaml")

petab_prob = PEtabODEProblem(model)

# Parameter estimation
using Optim, Plots
x0 = get_startguesses(petab_prob, 1)
res = calibrate(petab_prob, x0, IPNewton())
plot(res, petab_prob; linewidth = 2.0)
# Multistart optimization using 50 starts
ms_res = calibrate_multistart(petab_prob, IPNewton(), 50)
plot(ms_res; plot_type=:waterfall)
plot(ms_res, petab_prob; linewidth = 2.0)
```

Next steps:

- Explore different ODE solvers for your problem type
- Use gradient-based optimizers with automatic differentiation
- Perform uncertainty quantification with sampling methods

Documentation: https://sebapersson.github.io/PEtab.jl/stable/

Parameter Estimation with Data2Dynamics

Data2Dynamics (D2D) is a MATLAB-based framework for comprehensive modeling of biological processes with focus on ordinary differential equations.

Key features:

- MATLAB-based framework with PEtab support
- Profile likelihood-based uncertainty analysis
- Model identifiability analysis
- PEtab import functionality

Minimal working example:

```
% Setup Data2Dynamics environment
arInit;

% Import PEtab problem
arImportPEtab({'my_model','my_observables','my_measurements','my_conditions','my_parameters

% Multi-start optimization (100 starts)
arFitLHS(100);

% Display results
arPlotFits;
arPlot;
arPrint;
```

Documentation: https://github.com/Data2Dynamics/d2d/wiki

Contribute to the Benchmark Collection

Before diving into parameter estimation, consider contributing your PEtab problem to the community! The PEtab Benchmark Collection is a curated repository of parameter estimation problems that helps:

- **Validate** your PEtab problem by ensuring it works with multiple tools
- **Enable reproducibility** by providing a permanent reference for your model
- **Facilitate method comparison** by allowing others to test algorithms on your problem
- **Support the community** by expanding the available benchmark suite

How to contribute:

See their How to Contribute, and for a complete checklist see the pull request template.

Additional Resources

PEtab Ecosystem:

- PEtab Format Specification - Complete PEtab documentation
- PEtab Select - Model selection extension

Model Repositories:

- Benchmark Collection - Curated PEtab problems
- BioModels - Database of published SBML models

Getting Help:

- PEtab-GUI Issues: https://github.com/PEtab-dev/PEtab-GUI/issues
- PEtab Issues: https://github.com/PEtab-dev/PEtab/issues
- PEtab Discussion: https://github.com/PEtab-dev/PEtab/discussions
- Systems Biology Community: https://groups.google.com/g/sbml-discuss
